# Cutaneous Epithelial Tumors Induced by Vemurafenib Involve the MAPK and Pi3KCA Pathways but Not HPV nor HPyV Viral Infection

**DOI:** 10.1371/journal.pone.0110478

**Published:** 2014-10-31

**Authors:** Eric Frouin, Bernard Guillot, Marion Larrieux, Ariane Tempier, Nathalie Boulle, Vincent Foulongne, Céline Girard, Valérie Costes, Jérome Solassol

**Affiliations:** 1 Department of Biopathology, CHU Montpellier, Montpellier, France; 2 University of Montpellier I, Montpellier, France; 3 Department of Dermatology, CHU Montpellier, Montpellier, France; 4 U1058, INSERM, University of Montpellier I, Montpellier, France; 5 Department of Virology, CHU Montpellier, Montpellier, France; 6 Department of Clinical Oncoproteomics, Montpellier Cancer Institute (ICM), Montpellier, France; A*STAR, Singapore

## Abstract

The inhibitors of mutant BRAF that are used to treat metastatic melanoma induce squamoproliferative lesions. We conducted a prospective histopathological and molecular study on 27 skin lesions from 12 patients treated with vemurafenib. Mutation hot spots in *HRAS, NRAS, KRAS, BRAF*, and *Pi3KCA* were screened. HPV and HPyV infection status were also determined. The lesions consisted of 19 verrucal papillomas, 1 keratoacanthoma and 7 squamous cell carcinomas. No mutations were found within *BRAF* and *NRAS*. *KRAS*, *HRAS*, and *Pi3KCA* oncogenic mutations were found in 10 (83.3%), 7 (58.3%), and 4 (33.3%) patients respectively; however, these mutations were not consistent within all tumors of a given patient. *Pi3KCA* mutation was always associated with a mutation in *HRAS*. Finally, no correlation was found between the mutated gene or type of mutation and the type of cutaneous tumor or clinical response to vemurafenib. P16 protein level was not indicative of HPV infection. HPV was detected in only two lesions. Two cases had MCPyV, and one had HPyV7. In conclusion, neither HPV nor HPyV seem to be involved in the development of squamoproliferative lesions induced by verumafenib. By contrast, *HRAS* and *KRAS* play a predominant role in the physiopathology of these tumors.

## Introduction

Constitutive, activating mutations in the BRAF gene occur in nearly 66% of melanoma cases and result in a single amino acid substitution of valine for glutamic acid at residue 600 (V600E); less frequently, the valine is substituted by lysine (V600K) [1]. The selective BRAF inhibitor (BRAFi) vemurafenib is highly effective in treating metastatic melanomas and has been approved as a first-line therapeutic for metastatic melanoma cases that harbor V600 mutations in *BRAF* [2]. The skin is the most prevalent organ for side effects caused by BRAFi treatment. Squamoproliferative lesions, such as verrucous papillomas (VPs), keratoacanthomas (KAs) and squamous cell carcinomas (SCCs) occurred, respectively, in 79%, 14% and 26% of the patients [3]. VPs, also called verrucal keratosis or BRAFi-associated verrucous keratosis, are verruciform keratotic squamoproliferative lesions that resemble warts. Histologically, they present as well-differentiated epithelial lesions with cup-shaped architecture. Acanthosis, papillomatosis, hyperparakeratosis and hypergranulosis are usually characteristic of, but are not specific for this tumor type [4]. KA and SCC induced by BRAFi do not differ from those that are not caused by BRAFi, and they are invasive tumors. The MAPK pathway has been shown to be an important target for melanoma treatment, and its role in the development of non-melanoma skin cancer has been recently reported [5], [6]. In addition, despite histological similarities with verruca vulgaris, there are conflicting conclusions about the implication of viruses, especially Human Papilloma viruses (HPV), in the development of lesions induced by BRAFi [7]–[10]. Thus, to better understand the mechanisms by which skin tumors develop following BRAFi treatment, we conducted a histological, immunohistological and molecular study to evaluate both the MAPK pathway and the presence of HPV and other polyomaviruses (HPyV), especially Merkel cell polyomavirus (MCPyV), in a group of BRAFi induced VP, KA and SCC.

## Materials and Methods

### Patients and lesions

From July 2012 to March 2013, patients treated with vemurafenib who presented with lesions that had the clinical characteristics of SCC or KA (n = 12) were included in the present study. All lesions were excised and submitted to pathological examination. Briefly, skin samples were fixed in 4% formalin and embedded in paraffin. Hematoxylin and eosin (HE) stained sections were reviewed by two pathologists (EF and VC). Criteria that are described elsewhere [4] were used for the histopathological diagnosis of KA and VP, and the standard criteria were used for diagnosing SCC [11]. Some lesions displayed overlapping features, especially between VP and KA or SCC and KA, and these lesions were classified as VP or SCC if they did not satisfy the strict criteria for KA [4].

### Ethics statements

Patients were obtained from the department of Biopathology in protocols approved by the institutional review board of the University Hospital of Montpellier. The Investigators explained design and purpose of the study to participants. Potential participants were informed of their right to abstain from participation in the study or to withdraw consent to participate at any time without reprisal. We obtained a verbal informed consent statement from all individuals prior to their participation in the study in agreement with the University Hospital of Montpellier ethical review committee. Only verbal consent is relied on the French bioethics decree N° 2007–1220 published in the official journal of the French Republic. Consents were notified and recorded in the medical files at the University Hospital of Montpellier.

### Immunohistochemistry

Immunohistochemistry was performed using a Ventana-Benchmark-Ultra according to an ULTRAView universal DAB detection kit and procedure. P16 antibody (clone E6H4, Roche-Ventana) was applied for 32 minutes after epitope retrieval with CC1 (36 minutes). P16 immunostaining was scored as follows: 0 negative or fewer than 75% of the cells had positive nuclear and cytoplasmic staining and + in all other conditions.

### In situ Hybridization

ISH was performed with INFORM-HPVII-Family6 probe and INFORM-HPVIII-Family16 probe (Ventana-Medical-System) with an ISH iVIEW Blue Plus Detection Kit (Ventana-Medical-System) on a Ventana Benchmark Ultra in accordance with the manufacturer's instructions. A positive control was included on each slide.

### DNA isolation and mutation detection

Tumor-rich areas marked on the HE histologic sections were manually cored and collected in a microtube for genetic testing. Tumor genomic DNA was extracted from 2 to 3 punches using a Qiagen extraction kit (QIAamp-DNA FFPE tissue kit) according to the manufacturer's recommendations. DNA quantity and quality was measured using a NanoDrop1000.

Full coding sequences of 11 exons, including the oncogenetic mutational hot spots corresponding to the *KRAS* exon 2, *HRAS* exons 2 and 3, *NRAS* exons 2 and 3, *BRAF* exon 15, and *Pi3KCA* exons 1, 3, 4, 9 and 20, were analyzed. Sequencing of *KRAS*, *HRAS* and *NRAS* was performed by Sanger direct sequencing conducted after PCR amplification of targets exons on a 36-capillary 3130XL-DNA-Analyzer (Absciex). Table S1 summarizes the primer sequences used for Sanger direct sequencing. *BRAF* and *Pi3KCA* mutations were probed with allele-specific, real-time PCR on a CobasZ-4800 (Roche) and its associated software. All samples were analyzed in duplicate.

### HPV DNA detection

HPV DNA detection and typing was performed using the INNO-LiPA HPV Genotyping extra assay (Innogenetics) according to the manufacturer's instruction. The assay covers high-risk and probable high-risk HPV genotypes (16, 18, 26, 31, 33, 35, 39, 45, 51, 52, 53, 56, 58, 59, 66, 68, 73, and 82) as well as a number of low-risk HPV genotypes (6, 11, 40, 43, 44, 54, and 70) and some additional types (69, 71, and 74). HPV sequences were probed in sample extracts with two consensus PCR assays with primers PGMY09/11 for mucosal HPVs and primers FAP59/64 for cutaneous HPVs, as previously described [12], [13]. HPV detection was performed using 100 ng of tumor extracted DNA in each reaction.

### Genomic HPyV detection

MCPyV, HPyV6 and HPyV7 DNA sequences were detected by real-time PCR through 5' nuclease assays on a Lightcycler 480 apparatus using the LC480 probe master mix (Roche); previously described primers and probes targeting the respective VP3 coding region of each virus were used [14].

## Results

### Clinical and pathologic characterization of skin lesions

Twelve patients were included in the present study. Twenty-seven lesions were analyzed and classified as VPs (19 lesions, 70%), KA (1 lesion, 4%) and SCC (7 lesions, 26%). Seven patients developed more than one lesion, and 4 patients developed benign and malignant lesions. Ten patients developed a VP first, one developed a KA, and the final case developed SCC. Cutaneous tumors were developed within a median of 31 days after the start of treatment (range of 11 to 385 days) and the last epithelial lesion appeared after a mean of 6.2 months (2 to 13 months). Nearly all patients displayed an additional cutaneous side effect, especially photosensitivity, cutaneous drug rash and keratosis pilaris.

Primitive melanomas consisted of nodular melanoma in 3 cases, superficial spreading melanoma in 6 cases, and lentigo maligna melanoma in one case. The final 2 cases were not classified precisely. The Breslow index ranged from 0.7 to 17.52 mm (median 6.75 mm). Vemurafenib was the first line therapy for all patients and 2 had also undergone cerebral radiotherapy. All patients but one had a *BRAF* V600E mutation whereas the final patient instead had a V600K mutation. Because of disease progression or adverse events, vemurafenib was stopped in 6 patients after a mean of 5.2 months and no cutaneous epithelial lesions appeared after discontinuation of vemurafenib.

### Histopathological and immunohistopathological characterizations

**Figure 1:**
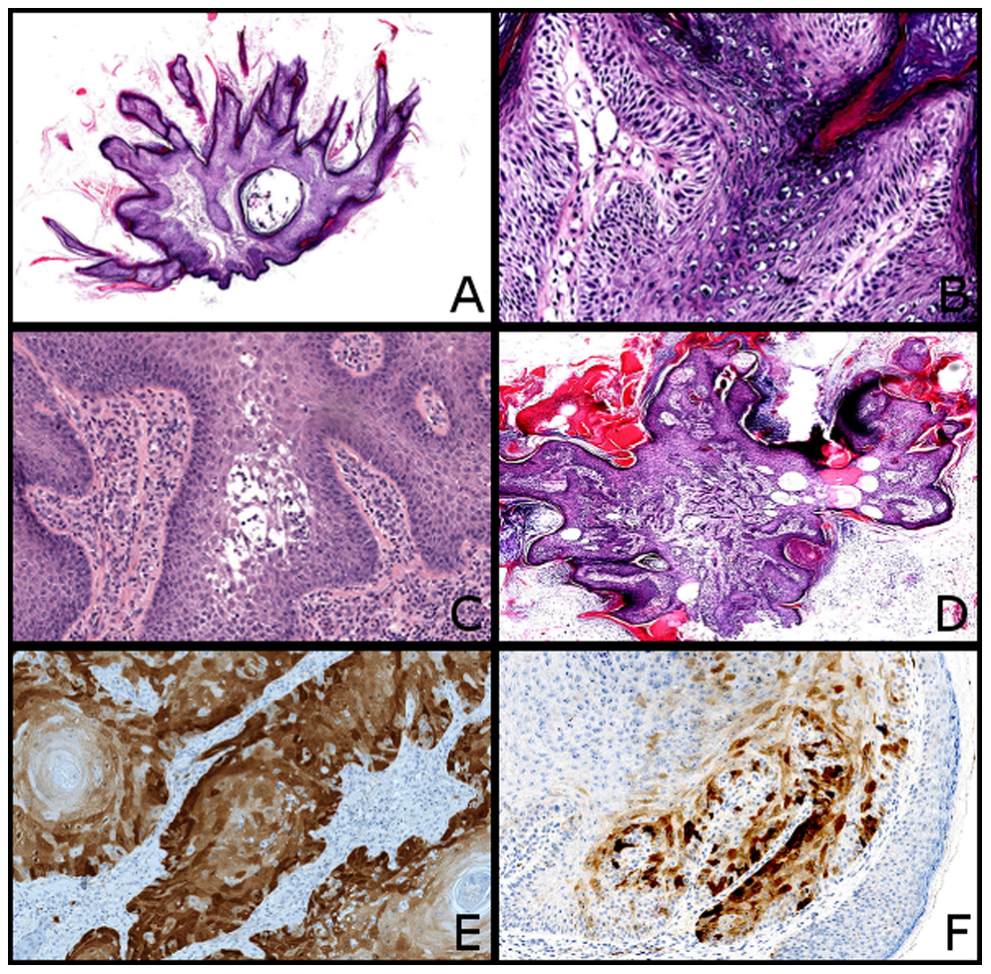
Histopathology and immunohistochemical findings of VP and SCC induced by vemurafenib. (A) Typical VP with verrucous and papillomatous architecture covered by hyperkertosis (HE, x20). (B) Note the preeminent granulomatous layer with clear keratinocytes suggestive of an HPV infection (HE, x200). (C) VP with acantholysis (HE, x100). (D) VP with invasion of the superficial dermis (HE, x20). (E) Strong P16 positivity in a SCC. This tumor did not have any HPV (x100). (F) Heterogenous P16 expression in a VP (x100).

Twenty-seven lesions were analyzed. VPs were verrucous (18 lesions) and papillomatous (16 lesions) (fig. 1.A). Hypergranulosis and clear keratinocytes within superficial portions were observed, respectively, in 19 and 5 VPs and were suggestive of a possible viral origin (fig. 1B). Two VPs displayed acantholysis (fig. 1C). Two VP were slightly invasive (fig.1D). KA was typical. SCCs were always well differentiated. Hypergranulosis and clear keratinocytes were observed in 4 and 3 lesions, respectively. No vascular or neural invasion was observed. None of the lesions recurred and none of the patients developed metastasis.

P16 was positive in 2 SCCs and 1 VP (fig. 1E). In 14 lesions, P16 was heterogeneously expressed, and the expression was mostly cytoplasmic (fig. 1F). There was no correlation between P16 expression and malignancy or invasion.

### Oncogenic mutations

The molecular results are summarized in table 1. For 7 tumors, PCR sequencing of *KRAS* and *Pi3KCA* was not possible due to DNA degradation. Mutations were found in all but one patient and in all but 2 lesions (2 VP). No mutations were found within the *BRAF* or *NRAS*. Twelve lesions (7 patients) had *KRAS* mutations. *KRAS* mutations were consistent in all lesions of a given patient. Fifteen tumors displayed *HRAS* mutations (9 VP, 1 KA and 5 SCC). Fifteen tumors displayed silenced mutations (9 VP, 1 KA and 5 SCC). The H27H polymorphism was consistent in all lesions of a given patient, except for in the case of 1 patient. Four tumors from four distinct patients had a *Pi3KCA* mutation (2 VP and 2 SCC). *Pi3KCA* was always associated with *HRAS* mutations and, in 2 cases, was also associated with *KRAS* mutations. Mutations in *HRAS*, *KRAS*, and *Pi3KCA* were not consistent within all tumors of a given patient and they were not correlated with benign or malignant behaviors, or clinical response to vemurafenib.

**Table 1:** Immunohistological and molecular analyses.

Patients	Lesion	HPV (ISH)	HPV (InnoLiPa)	HPV (consensus primers)	MCPyV	HPyV6	HPyV7	*BRAF*	*NRAS*	*HRAS*	*KRAS*	*Pi3KCA*
1	SCC	-	-	-	-	-	-	-	-	-	G12C	-
1	SCC	-	-	-	-	-	-	-	-	G13D/H27H	G12C	E542K
2	SCC	-	-	-	-	-	-	-	-	G12D	G12C	-
2	SCC	-	-	-	-	-	-	-	-	Q61L	G12C	-
2	VP	-	-	-	-	-	-	-	-	-	G12C	-
2	VP^1^	-	-	-	-	-	-	-	-	-	G12C	-
3	VP	-	-	-	-	-	-	-	-	Q61L	G12C	E545X
4	VP	-	-	-	-	-	-	-	-	-	G12C	-
5	VP	-	-	-	-	-	-	-	-	H27H	NI	-
5	VP	-	+^3^	-	-	-	-	-	-	H27H/Q61L	NI	-
5	VP^2^	-	-	-	-	-	-	-	-	H27H/F82L	-	-
6	VP	-	-	-	-	-	-	-	-	H27H/Q61L	G12C	-
6	SCC	-	-	-	-	-	-	-	-	H27H/Q21L	NI	E542K
7	VP	-	-	-	-	-	-	-	-	-	NI	-
8	VP^2^	-	-	-	-	-	-	-	-	G12D/K16K/H27H	G12C	-
8	VP	-	-	-	-	-	-	-	-	H27H	NI	-
8	KA	-	-	-	-	-	-	-	-	G12D/H27H	NI	-
8	SCC	-	-	-	-	-	-	-	-	H27H	G12C	-
9	VP	-	-	-	-	-	+	-	-	G12N	-	E542K
10	VP	-	-	-	-	-	-	-	-	Q61L	-	-
10	VP	-	HPV39	-	-	-	-	-	-	G12R	-	NI
10	VP	-	-	-	+	-	-	-	-	-	-	-
10	SCC	-	-	-	-	-	-	-	-	G12V	-	-
11	VP	-	-	-	-	-	-	-	-	H27H	-	-
11	VP	-	-	-	-	-	-	-	-	H27H	-	-
11	VP	-	-	-	-	-	-	-	-	H27H/Q61L	-	-
12	VP	-	-	-	+	-	-	-	-	H27H	G12D	-

1 One VP had a histological appearance of possible regressive keratoacanthoma,

2 while 2 VPs displayed slightly invasion.

3 For one sample, HPV was detected by InnoLipa and could not be further characterized. -: no mutation or HPV, MCPyV, HPyV6 or HPyV7 was detected within the sample. NI (not informative): for 7 tumors, no results were obtained for *KRAS* or *Pi3KCA* sequencing.

### Viral findings

In situ hybridization for HPV was consistently negative. HPV39 and an uncharacterized HPV (HPV X) were found in 2 VP. However, the presence of these HPV types was not confirmed after PCR by other consensus PCR assay. MCPyV was detected in 2 VP and HPyV7 in another VP.

## Discussion

The development of VP, keratoacanthomas and SCC in patients taking BRAF inhibitors has been thoroughly described [3], [4], [15]. Squamoproliferative lesions are classified as an early side effect of BRAFi, appearing within the first 3 to 6 weeks after treatment initiation [15], [16]. In our study, cutaneous lesions developed after a median duration of 31 days; however, later onset of the first cutaneous tumor was also observed in 3 patients (mean 228 days). Interestingly, the squamoproliferative lesions were clearly related to vemurafenib therapy, as no lesion appeared after the discontinuation of the treatment. In addition, no skin lesions regressed after the discontinuation of vemurafenib.

Despite histopathological findings, HPV was only detected in our study in 2 VPs (7.4% of lesions) and in 2 different patients by two independent approaches, including in situ hybridization with 2 probe sets. One VP sample exhibited a non-typable HPV and the other one was HPV39-positive, a genotype that is usually present in the mucous epithelia but not in skin. HPV39 is classified within the HPV high-risk group and induces epidermoid carcinomas instead of benign lesions. However, the other lesions from the same respective patient did not exhibit this HPV subtype. This result is consistent with recent studies suggesting that HPVs are unlikely to be a contributor to VP or SCC tumors. Using next generation sequencing, Ganzenmueller et al. could not find HPV in any of the 5 patients with vemurafenib -associated verrucous keratosis lesions (and approximately 20 lesions by patients) [10]. Anforth et al. screened HPV in a cohort of six and 10 vemurafenib-induced SCCs and verrucal keratosis, respectively. HPV was positive in one verrucal keratosis but was negative in all other tissue samples [5]. In a very recent study, Holderfield et al. analyzed 62 cutaneous lesions from 44 vemurafenib-treated patients, including benign and malignant tumors, for HPV expression. The authors reported that HPV was present in only a subset (13%) of samples [17]. Six HPV subtypes were observed: HPV9 and HPV38 (twice for each of them), HPV32, HPV49, HPV80, and HVP100 (once for each of them). However, authors also observed that vemurafenib could induce SCC tumorigenesis in K14-HPV16 mice whereas HPV negative, non-transgenic littermates did not. Moreover, vemurafenib-induced SCCs were found in both RAS wilt-type and RAS mutated samples allowing authors to propose that vemurafenib cooperates with HPV in human patients to promote SCC's initiation in either the presence or absence of RAS mutations [17]. Such discrepancy or apparently contradiction could likely be explained by differences between mice tumorigenesis models and patients. Papillomaviruses are strictly species-specific and HPV-transgenic mice, if of interest for carcinogenicity studies, remain restricted models of HPV infection. K14-HPV16 mice (that express the early region of HPV16 E6-E7 in basal cells of the squamous epithelium under the control of the K14 promoter) backcrossed into the FVB/n background develop dysplastic lesions that systematically progress to SCCs whereas K14-HPV16 mice with C57BL/6 or BALB/c genetic backgrounds develop hyperplastic and/or dysplastic lesions [18]. Occurrence of vemurafenib-induced SCC lesions in this transgenic model could likely be driven by specific or differential processes than those triggered by the natural HPV16 infection. In addition, epidemiological studies have clearly demonstrated that cutaneous SCCs in patients are associated to diverse HPV types such as HPV8 (β-HPV) and vemurafenib-induced SCC lesions frequently harbour multiple HPV types in a single sample [18]–[20]. Finally, authors found KRAS mutations in induced tumors while several clinical data demonstrated that HRAS mutations widely predominate in vemurafenib-induced tumors [5]. Altogether, these data demonstrated that the potential implication of HPV in the development of cutaneous epidermal remains unclear.

In our study, no mutations were found in the BRAF and NRAS in squamo-proliferative lesions induced by vemurafenib. In contrast, HRAS and KRAS appeared mutated in a total of 21 lesions (77%), in 15 (55%) and 12 (44%) lesions, respectively. Previously published studies found less frequent mutations in the RAS genes in BRAFi induced lesions (50% in Anforth et al's and 57% in Su et al's) [5], [16]. The most prevalent mutations were encountered at codon 61. We describe herein new mutations in *HRAS* (G13D and F82L). The development of SCC and VP with BRAFi has been thoroughly demonstrated in several experimental models using various read-out methods. The prevalent hypothesis is that the binding of RAF inhibitors to BRAF induces RAS-dependent BRAF/CRAF dimerization and activation of the pathway transmitted by CRAF [21], [22]. In this model, the cells are wild type for *BRAF* and harbor an activated RAS. Another mechanism involves an activated RAS (mutated or activated upstream by EGFR). Vemurafenib binds to one member of RAF homodimers (CRAF/CRAF) or heterodimers (BRAF/CRAF) and transactivates the other element of the dimer [23]. In this latter model, activation is dependent on the dose of the RAF inhibitor with activation of the pathway at a low concentration and inhibition at a higher concentration. Increased levels of phospho-ERK in a wild type *BRAF* cell line treated with vemurafenib further supports this theory [23]. Actinic keratosis and SCCs have been shown to harbor mutations in *RAS* genes and in *TP53*, but these mutations are less frequent than in squamoproliferative lesions induced by BRAFi (3.2% vs 21.1%) [6]. To the best of our knowledge, our study is the first to show that tumors with similar histopathological features arising in a single patient treated with BRAFi could harbor distinct mutations in one or multiple *RAS* genes. Interestingly, we observed mutations in 2 distinct *RAS* genes in 6 lesions, a finding that has not been previously reported. However, these mutations do not seem to have any implications in aggressive behavior.

In our study, *Pi3KCA* was mutated in 4 lesions (15%) and was always associated with mutations in *RAS* genes. Anforth et al reported similar results with the detection of 4 mutations in the *Pi3KCA* gene in 19 lesions (21%), although in this report, *Pi3KCA* gene mutations were unique in 3 cases [5]. Oberholzer et al have shown that SCCs and actinic keratosis may harbor mutations in the *Pi3KCA* gene. In their most recent study on this topic, mutations in the *Pi3KCA* gene were found in patients treated by rapamycin and sirolimus or patients who developed SCCs and AKs but not in patients treated by BRAFi (vemurafenib) [6]. Hotspot mutations were present in amino acids 542, 545 and 1047 [5], [6]. As in RAS genes, these mutations were present prior to BRAFi; however, their roles have yet to be clearly defined.

Finally, we observed a frequent polymorphism in *HRAS* (H27H) in 6 patients (50%). This polymorphism has been described in different populations such as Chinese, Indian, and German, but it has never been reported in the French population [24]–[26]. In these studied populations, variant allele (TC and CC) occurs with a frequency of 16% to 40% of controls. In addition, *HRAS* H27H polymorphism was statistically associated with the risk of developing various cancers (gastric, thyroid, bladder, and oral squamous cell carcinoma) and was detected both in tumor and in blood samples [24]–[28]. In addition, it has been reported in a SCC lesion of one patient treated with dabrafenib [8]. In contrast to other *HRAS* mutations, this polymorphism may only reflect cancer susceptibility.

In conclusion, despite limited number of tested patients and the geographically restrictive location of our cohort, our results are indicative of a probably low implication of HPV in the development of squamoproliferative lesions induced by vemurafenib. The expression of HPyVs in 2 lesions could be explained by their high frequency in the skin [29]. Further investigations are needed to conclude on the possible implication of β-HPV in vemurafenib-induced epithelial lesions.

## Supporting Information

Table S1 The primer sequences used for Sanger direct sequencing of the *KRAS*, *HRAS* and *NRAS*.
(DOC)
