# Supplementary material for: Cutaneous Epithelial Tumors Induced by Vemurafenib Involve the MAPK and Pi3KCA Pathways but Not HPV nor HPyV Viral Infection
Source: PLoS One. 2014 Oct 31;9(10):e110478. doi: 10.1371/journal.pone.0110478 (PMC4215900; doi:10.1371/journal.pone.0110478)
Supplement: Table S1 — The primer sequences used for Sanger direct sequencing of the KRAS, HRAS and NRAS. (DOC) [file pone.0110478.s001.doc]

**Supplementary Table 1:** The primer sequences used for Sanger direct sequencing of the *KRAS*, *HRAS* and *NRAS*.

| **Gene / exon** | **Primer sequences** |
| --- | --- |
| *KRAS* / 2 | F : 5’-TAACCTTATGTGTGACATGTTCT-3’  R : 5’-TGGTCCTGCACCAGTAATA-3’ |
| *HRAS* / 2 | F : 5’-CAGGAGACCCTGTAGGAGGA-3’  R : 5’-CAGGACACAGCCAGGATAGG-3’ |
| *HRAS* / 3 | F : 5’-GGAAGCAGGTGGTCATTGAT-3’  R : 5’-AGTACAGGTGAACCCCGTGA-3’ |
| *NRAS* / 2 | F : 5’-GATGTGGCTCGCCAATTAAC-3’  R : 5’-CACTGGGCCTCACCTCTATG-3’ |
| *NRAS* / 3 | F : 5’-CACACCCCCAGGATTCTTAC-3’  R : 5’-GCTTCCTCTGTGTATTTGCCA-3’ |
